# Intelligent contour extraction approach for accurate segmentation of medical ultrasound images

**DOI:** 10.3389/fphys.2023.1177351

**Published:** 2023-08-22

**Authors:** Tao Peng, Yiyun Wu, Yidong Gu, Daqiang Xu, Caishan Wang, Quan Li, Jing Cai

**Affiliations:** ^1^ School of Future Science and Engineering, Soochow University, Suzhou, China; ^2^ Department of Health Technology and Informatics, The Hong Kong Polytechnic University, Kowloon, Hong Kong SAR, China; ^3^ Department of Radiation Oncology, UT Southwestern Medical Center, Dallas, TX, United States; ^4^ Department of Ultrasound, Jiangsu Province Hospital of Chinese Medicine, Nanjing, Jiangsu, China; ^5^ Department of Medical Ultrasound, The Affiliated Suzhou Hospital of Nanjing Medical University, Suzhou Municipal Hospital, Suzhou, Jiangsu, China; ^6^ Department of Radiology, The Affiliated Suzhou Hospital of Nanjing Medical University, Suzhou Municipal Hospital, Suzhou, Jiangsu, China; ^7^ Department of Ultrasound, The Second Affiliated Hospital of Soochow University, Suzhou, China; ^8^ Center of Stomatology, The Second Affiliated Hospital of Soochow University, Suzhou, China

**Keywords:** medical image segmentation, ultrasound image, adaptive selection principal curve, quantum evolution neural network, explicable mathematical formula

## Abstract

**Introduction:** Accurate contour extraction in ultrasound images is of great interest for image-guided organ interventions and disease diagnosis. Nevertheless, it remains a problematic issue owing to the missing or ambiguous outline between organs (i.e., prostate and kidney) and surrounding tissues, the appearance of shadow artifacts, and the large variability in the shape of organs.

**Methods:** To address these issues, we devised a method that includes four stages. In the first stage, the data sequence is acquired using an improved adaptive selection principal curve method, in which a limited number of radiologist defined data points are adopted as the prior. The second stage then uses an enhanced quantum evolution network to help acquire the optimal neural network. The third stage involves increasing the precision of the experimental outcomes after training the neural network, while using the data sequence as the input. In the final stage, the contour is smoothed using an explicable mathematical formula explained by the model parameters of the neural network.

**Results:** Our experiments showed that our approach outperformed other current methods, including hybrid and Transformer-based deep-learning methods, achieving an average Dice similarity coefficient, Jaccard similarity coefficient, and accuracy of 95.7 ± 2.4%, 94.6 ± 2.6%, and 95.3 ± 2.6%, respectively.

**Discussion:** This work develops an intelligent contour extraction approach on ultrasound images. Our approach obtained more satisfactory outcome compared with recent state-of-the-art approaches . The knowledge of precise boundaries of the organ is significant for the conservation of risk structures. Our developed approach has the potential to enhance disease diagnosis and therapeutic outcomes.

## 1 Introduction

Medical image segmentation techniques have been essential for the early diagnosis of clinical disease. They have primarily been used to discover the region of interest (ROI) in medical images. Due to its ability to generate real-time images and its low cost, ultrasound imaging has been one of the most commonly used imaging techniques for early disease detection. However, the precise segmentation of organs in ultrasound images remains challenging, as 1) the boundaries of organs (i.e., prostate and kidney) are ambiguous or have unseen regions owing to the low contrast of ultrasound images, and 2) the shapes of organs vary between different patients.

Medical image segmentation has become a considerable research field. Lei *et al*. (Lei et al., 2021) devised an improved deep convolution network to segment multiple organs (i.e., bladder, prostate, rectum, and urethra) in the male pelvic region, where an anchor-free-based module assisted the proposed model to precisely capture the relationship of both locations and shapes among multiple organs. However, the capability of this technique depends on the number of training images. In addition, a validation set was not used, so the selection of the hyper-parameters (i.e., learning rate and optimal epoch) of the model was not allowed. Due to the limited amount of ultrasound data for training, Amiri *et al*. (Amiri et al., 2020) used the pre-trained Unet model for ultrasound image segmentation. The pre-trained Unet model was trained on the XPIE dataset (Xia et al., 2017), which includes 10,000 natural images. In addition, the newly added deep and shallow layers can potentially be used to search for the best scheme for the transfer learning model. However, the final segmentation performance is affected by the correlation between the two datasets. In the ultrasound image segmentation task, Xu *et al*. (Xu et al., 2021) added a vector-based attention layer to the convolutional neural network to balance spatial and channel attention so that it can better highlight the salient features. In addition, the geometric priors of specific organs were used to improve the accuracy of detection. However, a validation set was not used for the estimation of hyper-parameters in the learning algorithms. Among the different types of segmentation methods, contour extraction methods are known for their ability to extract realistic shapes of organs in medical images.

A region expression or curve description model has been the primary goal of the contour extraction models developed to express realistic contours of tissues. Zhang *et al*. (Zhang et al., 2020) designed a multiple-channel, atrous-based neural network (NN) to segment ultrasound slices. In this model, the multiple-channel convolution layer and the atrous-based module were used to accurately collect multi-scale knowledge. Mishra *et al*. (Mishra et al., 2019) developed a deeply supervised network for segmentation in ultrasound images, in which a fusion layer was used to improve the flexibility of the network so that it could automatically choose the best features for further refinement. However, the accuracy of the network was influenced by the size of the input images. He *et al*. (He et al., 2021) proposed a synergistic image-level and voxel-level segmentation network, where the model used a contour-aware module for sampling to potentially increase the precision of delineation of the prostate outline. Panigrahi *et al*. (Panigrahi et al., 2019) used the multi-scale Gaussian fuzzy clustering algorithm to roughly segment the ROIs in ultrasound images. A multi-scale vector field convolution algorithm was then used to fine-tune the accuracy of the ROI. However, too many referred parameters of the proposed network were potentially required to be manually initialized. Liu *et al*. (Liu et al., 2021) introduced threshold segmentation into a deep-learning framework to generate the S-Mask R-CNN + Inception-v3 model to detect disease, but the Dice similarity coefficient (DSC) (Peng et al., 2018a) of the testing results was approximately 0.87.

In this work, we summarize the technical contributions of our segmentation approach in the ultrasound image segmentation field, as described below:

1) As the accurate contour extraction of organs in ultrasound images is a difficult task, the DSCs of fully-automatic methods are approximately 0.9 (Girum et al., 2020; Wang et al., 2019). Therefore, here, we present a semi-automatic contour extraction framework using radiologist-defined data points as the prior, resulting in a DSC of 0.957.
2) Due to their satisfactory performance at handling noisy input, principal curve (PC) techniques are widely adopted for distinguishing abnormal tissues from other surrounding regular tissues (Peng et al., 2018a). However, the number of vertices needs to be pre-determined by the users. Our method proposed herein addresses this problem.
3) As the contours of PC-based techniques consisting of segments are not smooth (Biau and Fischer, 2012), our method was developed, which used an interpretable mathematical formula to smooth the experimental contour.

In summary, the detailed advantages of the proposed approach compared with current approaches are as follows:

1) Differing from standard PC-based methods (Peng et al., 2018a), the adaptive selection principal curve (ASPC) method combines the neutrosophic-set-based mean shift (NSMS) method with the PC-based projection step. The advantage of the ASPC method is that it automatically determines the number of cluster vertices and then obtains the data sequence.
2) Differing from the mean shift clustering (MSC) method (Cheng, 1995), our NSMS method was able to achieve more robust outcomes, as it includes the natural capability of the neutrosophic set (NS) to study the neutralities’ nature to handle indeterminate information, especially noise, well.
3) To the best of our knowledge, the memory-based quantum-inspired differential evolution (MQDE) method is the first attempt to facilitate acquiring an optimal fractional-order backpropagation neural network (FBNNL) (Chen et al., 2020). Differing from the quantum-inspired differential evolution (QDE) technique, both a memory-based mechanism (Peng et al., 2021) and the Cuckoo search algorithm (Cobos et al., 2014) were used while innovatively designing a new mutation technique to enhance the ability of the model to handle different types of multimodal issues and including the newly proposed global optimum scheme to acquire the appropriate parameters of the model.
4) Due to the excellent storage and heredity ability of the Caputo-derivative-based fractional gradient descent algorithm, we used the FBNNL (Chen et al., 2020). In addition, we used the exponential linear unit (ELU) function (Bernal et al., 2019) to take the place of the sigmoid function (Bernal et al., 2019) that we used in our previous study (Peng et al., 2018a) to address the vanishing gradients issue.
5) To smooth the contours of PC-based methods, we used an interpretable mathematical model to express the smooth organ contour, which is denoted by the parameters of the optimized FBNNL.

A previous study (Peng et al., 2022a) (namely, *H-SegMed* method) is related to this current study; however, there are some differences between the two studies, as indicated below.

1) Compared with the previous study (Peng et al., 2022a), here we used multiple datasets, including prostate and kidney datasets, rather than only one prostate dataset to evaluate the performance of our model.
2) Here, we innovatively used the ASPC method to automatically decide the number of vertices/clusters, whereas this required human intervention in the previous study (Peng et al., 2022a).
3) Here, we newly integrated the quantum computing characteristics into the evolution NN to enhance the capability of searching between global and local, while innovatively adding the Cuckoo search algorithm (Cobos et al., 2014) to improve the ability to select the optimal parameters.
4) Here, we adopted the ELU activation function (Bernal et al., 2019) to substitute the Tanh activation function used in the previous study (Peng et al., 2022a) to handle the gradient vanishing problem that appeared before. Based on this change, we developed an ELU-based mathematical model of organ contour using the mathematical derivation process.

## 2 Materials and methods

### 2.1 Problem formulation

**FIGURE 1:**
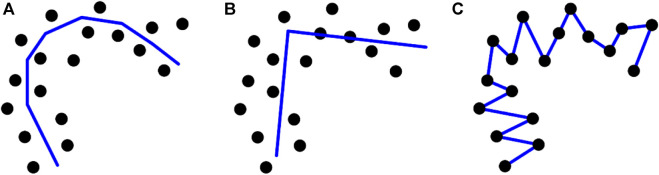
Different numbers of segments cause different achievements when using PC-based models. **(A)** an appropriate number of segments (*k*), **(B)** too few segments, and **(C)** too many segments.

Due to the existence of strong artifacts, there are ambiguous or unseen organ regions in ultrasound images, which makes it challenging to find the ultrasound organ contour. The DSCs of most automatic methods (Girum et al., 2020; Wang et al., 2019) are approximately 0.9. To improve the segmentation accuracy, we designed a point-guided segmentation model using a few points as the prior. Several researchers have used the PC method to identify a PC that can express the general direction of the data (Kégl et al., 2000). However, the performance of the PC-based method, which is affected by the number of segments, is always variable (Biau and Fischer, 2012; Peng et al., 2021) (shown in Figure 1), as the number of cluster vertices is pre-decided by the users (Moraes et al., 2020). Hence, pre-setting the number of vertices/clusters is critical for the results of PC-based methods. Moreover, the outcomes of PC-based techniques are composed of segments (Biau and Fischer, 2012) (shown in Figure 1), and smoothing the results becomes an important issue.

### 2.2 Detection model

**FIGURE 2:**
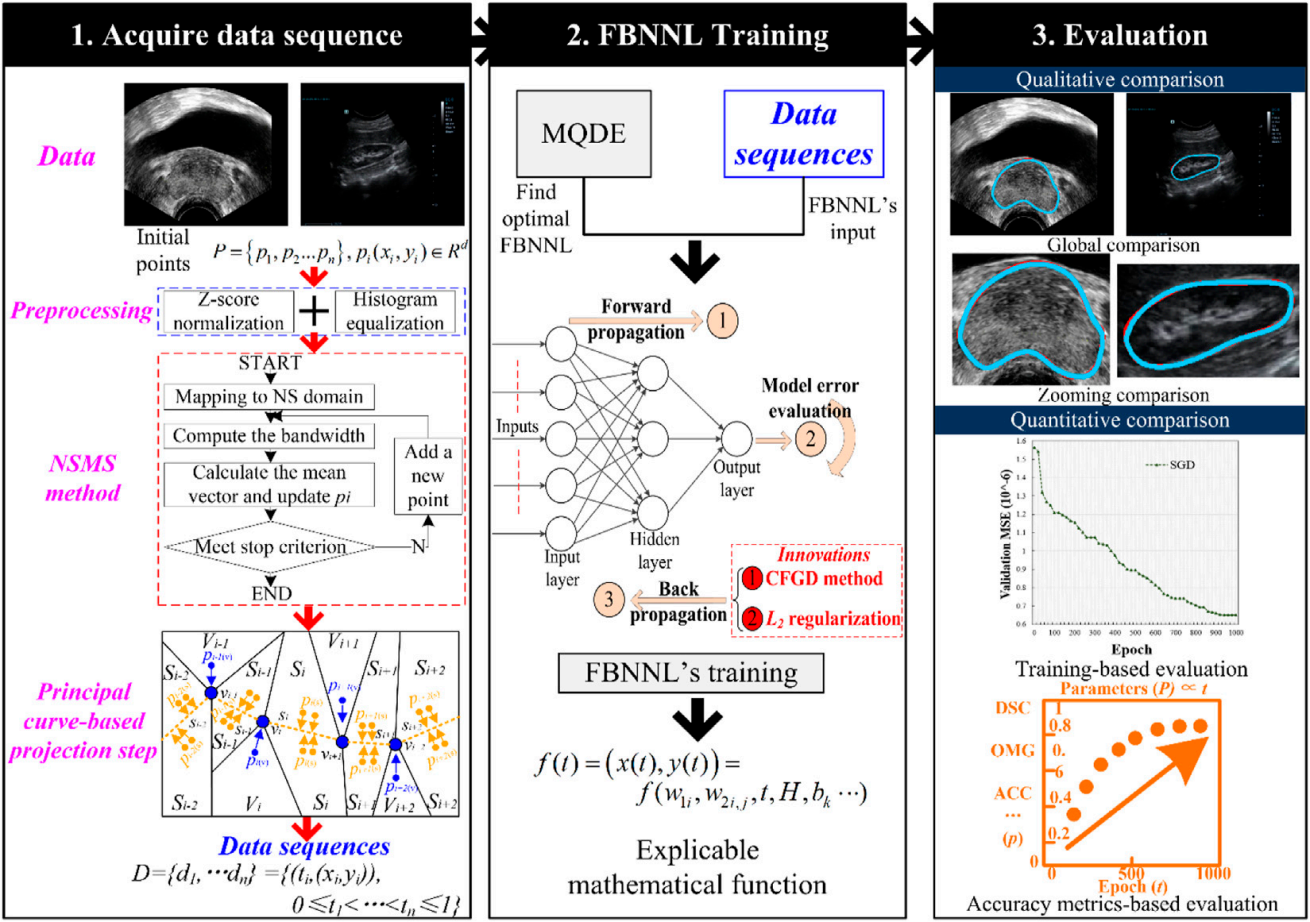
Design of our method. The first stage is to acquire a data sequence via the PC-based method. The second stage is to obtain the smooth and interpretable organ contour during the evolution-based neural network’s training. After training, the qualitative and quantitative evaluation is adopted for the experimental results in the third stage. Here are the abbreviations used in the figure: NSMS: neutrosophic-set-based mean shift method; MQDE: memory-based quantum-inspired differential evolution method; FBNNL: fractional-order backpropagation neural network; CFGD: Caputo-type fractional gradient descent method; PC: principal curve; DSC: Dice similarity coefficient; OMG: Jaccard similarity coefficient; and ACC: accuracy.

For accurate contour detection, we developed a hybrid segmentation method for medical ultrasound images. The method contains four main stages. In ***Stage 1***, we adopted the ASPC model to achieve the data sequence *D*, where *D* includes the data point *p*_*i*_ and the corresponding projection index *t*. In ***Stage 2***, the MQDE was designed to achieve the initial optimal parameters (i.e., weights and thresholds) of the FBNNL. In ***Stage 3***, the projection index *t* was used as the determined FBNNL input, and the coordinates of *p*_*i*_ were used as the expected outcome values for computing the global model error *E*. During the training of the FBNNL, the FBNNL’s model deviation *E* decreased, and the optimal FBNNL was obtained. In ***Stage 4***, an interpretable mathematical model of the organ contour was used to smooth the results. This was expressed by the model parameters of the optimal FBNNL. Figure 2 presents the design of our method.

#### 2.2.1 Stage 1: acquire the data sequence

**FIGURE 3:**
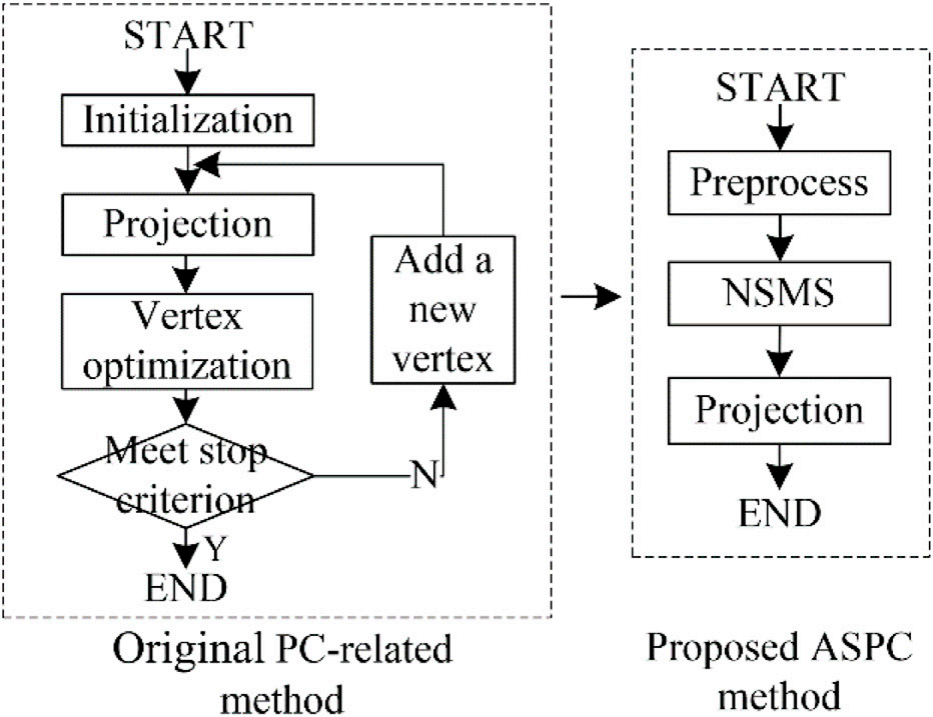
Difference between the original PC-based method (Peng et al., 2018a; Wu et al., 2021) and the proposed adaptive selection principal curve method. Here are the abbreviations used in the figure: PC: principal curve; NSMS: neutrosophic-set-based mean shift method; ASPC: adaptive selection principal curve.

We designed the ASPC method to acquire the data sequence. This method combines the NSMS algorithm with the PC-based projection step. Compared with standard PC-based methods (Peng et al., 2018a; Wu et al., 2021), the main improvement of the proposed ASPC method is the automatic determination of the vertices/clusters of the PC. Figure 3 shows a comparison between the standard PC-based method and our ASPC method.

##### 2.2.1.1 Preprocessing stage

We used Z-score normalization (Kabir et al., 2015) as the outlier prevention preprocessing technique to detect and remove outliers, while histogram equalization (Stark, 2000) was used to enhance the contrast in the images.

##### 2.2.1.2 NSMS method

Cheng *et al*. (Cheng, 1995) developed the traditional MSC method to search for the data cluster. However, many previous studies have demonstrated that the MSC method, with adaptive bandwidth, can generate more accurate results than the fixed-bandwidth module (Comaniciu et al., 2001). Furthermore, image noise, which is one type of uncertain information, may affect segmentation accuracy (Nguyen et al., 2019). NS has an inherent ability to study the neutralities’ ability to handle uncertain information, especially noise, well (Nguyen et al., 2019). Hence, we introduced an improved NS-related filter into the MSC method. The workflow of the NSMS method is described below.

Step 1: Map the original data set *P*_*n*_ into the channels in the neutrosophic set, in which *Tc*(*P*_*n*_), *Ic*(*P*_*n*_), and Fc(*P*_*n*_) indicate the light, indeterminate, and non-light data pixel sets, respectively.

$$T_{C}\left(x,y\right)=\frac{g\left(x,y\right)+g_{\min}}{g_{\max}-g_{\min}}$$ (1)

$$I_{C}\left(x,y\right)=\frac{Gd\left(x,y\right)+Gd_{\min}}{Gd_{\max}-Gd_{\min}}$$ (2)

and

$$F_{C}\left(x,y\right)=\frac{g_{\max}-g\left(x,y\right)}{g_{\max}-g_{\min}}$$ (3)

where *g*(*x*, *y*) indicates the intensity value and *Gd*(*x*, *y*) indicates the gradient value in the location *p*(*x*, *y*).

Step 2: Compute all of the channels according to the uncertain filter.

$$\sigma_{I}\left(x,y\right)=aI_{C}\left(x,y\right)+b$$ (4)

and

$$G_{Ic}\left(u,v\right)=\frac{1}{2\pi \sigma_{I}^{2}}\exp\left(-\frac{u^{2}+v^{2}}{2\sigma_{I}^{2}}\right)$$ (5)

where $\sigma_{I}$ represents the standard deviation that fixes the feature of the kernel function, and *G*_*Ic*_ represents the kernel function of the indeterminacy filter.

Step 3: Calculate the uncertain outcomes of the channels in the neutrosophic set

$$Tc^{\prime }\left(x,y\right)=\sum_{v=y-m/2}^{y+m/2}\sum_{u=x-m/2}^{x+m/2}Tc\left(x-u,y-v\right)G_{Ic}\left(u,v\right)$$ (6)

where *Tc’*represents the outcome using an uncertain filter on *Tc*, and *m* represents the size of the filter.

Step 4: Use the randomly picked ungrouped point *p*_*i*_ to calculate the bandwidth *h*.

$$h\left(x,y\right)=Ic_{avg}\left(x,y\right)\left(\max\left(Tc^{\prime }\right)-\min\left(Tc^{\prime }\right)\right)$$ (7)

where *Ic*_*avg*_ shows the mean uncertain value of the recent cluster point.

Step 5: Calculate the mean shift vector *m*(*p*).

$$m\left(p\right)=\frac{\sum_{i=1}^{n}p_{i}\times L\left({\left\|\frac{p-p_{i}}{h}\right\|}^{2}\right)}{\sum_{i=1}^{n}L\left({\left\|\frac{p-p_{i}}{h}\right\|}^{2}\right)}-p$$ (8)

Step 6: Transfer *p*_*i*_ in the direction *m*(*p*), and it obeys the rule that *p*_*i*_ = *p*_*i*_ + *m*(*p*).

Step 7: Jump to Step 5 when stop condition $\nabla f\left(p\right)=0$ is satisfied.

Step 8: Use the average value of the current cluster in uncertain domain to compute the bandwidth *h*.

Step 9: Jump to Step 5 when the current cluster point is stable.

Step 10: Jump to Step 4 when all data points are classified.

Step 11: The cluster points are achieved after the loop ends.

##### 2.2.1.3 The PC-based projection step

**FIGURE 4:**
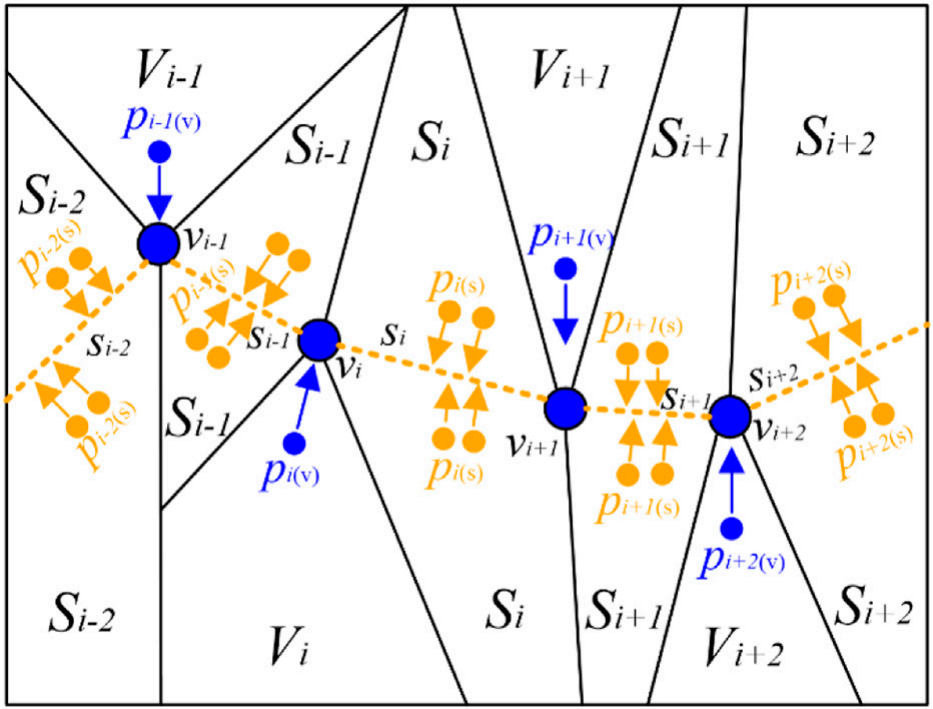
Optimal partition results according to the vertices and segments of the PC. For better visualization, we set the color of points *p*_*i*_ projecting to vertices *v*_*i*_ as blue and those projecting to segments *s*_*i*_ as orange. PC: principal curve.

After completing the NSMS method, we obtained the vertices/clusters and then determined the PC *f*. Hastie *et al*. (Hastie and Stuetzle, 1989) first proposed a “self-consistent” PC passing through the “middle” of the data cloud. We assumed that *f* shows a polygon connected with vertices *v* and line segments *s*. During the PC-based projection step, we scanned the PC *f* near the data point *p*_*i*_, projected *p*_*i*_ to the nearest neighborhood sets (vertices set *V*_*i*_ or segments set *S*_*i*_), and then acquired the projection index *t* (Kégl et al., 2000) of *p*_*i*_. The corresponding projection indices of the remaining points were also obtained using this method. Figure 4 shows the partition results according to the vertices and segments of the PC.

#### 2.2.2 Stage 2: finding the optimal NN

As the NN is easily trapped into the local minimum during training, the MQDE method was used to help search for the optimal initial FBNNL.

##### 2.2.2.1 Improvements of our MQDE method

There are drawbacks to the use of gradient-optimization-based NNs due to the trend of being trapped into the local optimum. We designed an MQDE method to search for the best configuration variables (i.e., weights and thresholds) of the NN. Differing from the QDE method (Draa et al., 2011), we developed the MQDE method by including some improvements, such as 1) a *memory-based mechanism* (Peng et al., 2021), 2) a *new mutation technique*, 3) the *Cuckoo search* (*CS*) *technique*, and 4) a *global optimum scheme*. The pseudo-code of the basic QDE method (Draa et al., 2011) is presented in Supplementary Appendix S1.

1) *Memory-based mechanism* (Peng et al., 2021): The purpose of this mechanism is to deposit the optimal average mutation factor (uF) and crossover rate (uCR) from the last iteration and use them as the initialization of the next iteration. The architecture of the memory-based MQDE method is presented in this section.
2) *New quantum mutation method*: We have listed some well-known mutation strategies of the QDE method in Supplementary Appendix S2. These strategies are appropriate for dealing with different optimization issues. DE/rand/1 and DE/rand/2 are known to focus on exploration; thus, they are suitable for dealing with multimodal issues. Meanwhile, both DE/best/1 and DE/best/2 focus on exploitation and are therefore appropriate for handling unimodal issues (Li et al., 2016; Cui et al., 2018). Hence, we developed a new quantum mutation scheme named DE/superior/1, which combines the advantages of the DE/rand/1 and DE/best/1 quantum mutation schemes, as shown by:

$$vec_{i}^{g}=\alpha_{base}^{g}+F\times \left(\alpha_{i2}^{g}-\alpha_{i3}^{g}\right)$$ (9)

and

$$\alpha_{base}^{g}=\lambda \times \alpha_{i1}^{g}+\left(1-\lambda \right)\times \alpha_{\sup erior}^{g}$$ (10)

where integers *i*_*1*_, *i*_*2*_, *i*_*3*_ are randomly selected within [1, *NP*], and are different from *i*. *α* ^*g*^_*superior*_ is randomly chosen from the superior individuals randomly, including the top math.floor(α*NI) individuals in the current population. math.floor(*α*) is a rounding function, which returns the largest integer not greater than its argument *α*. The base vector *α*_*base*_ is influenced by the adjustment parameter λ, where *α*_*base*_ is close to a randomly selected superior individual. Different values of λ determine the selection of different quantum mutation schemes. If λ = 1, DE/superior/1 tends to be DE/rand/1, and if λ = 0, DE/rand-superior/1 tends to be DE/superior/1. Hence, the selection of λ balances both exploration performance and exploitation performance of the quantum mutation scheme.

$$\lambda ={\left(\frac{g_{\max}-g}{g_{\max}}\right)}^{2}$$ (11)

3) *CS method* (Cobos et al., 2014): The CS method is a nature-inspired algorithm that is commonly used to find ROIs. During the process of the CS method, all candidates were randomly obtained. Let the *i-th* solution in the *(g+1)*-t*h* generation be *α*_*base*_^*g+1*^, and a Levy flight is performed as follows:

$$\alpha_{base}^{g}=\alpha_{base}^{g-1}+Levy\left(ap\right)$$ (12)

where *ap* is the adjusted parameter. Levy flight essentially supplies a random walk when random steps are drawn from a Levy distribution for a big step.

$$Levy\left(ap\right)=g^{-ap},1\leq ap\leq 3$$ (13)

When combining the CS method (Eqs. 12, 13) into the new quantum mutation method of the MQDE method (Eqs. 9, 10), a new mutant vector *nvec*_*i*_^*g*^ can be generated in Eq. 21.

$$nvec_{i}^{g}=rand\left[0,1\right]\times \alpha_{base}^{g}+\left(1-rand\left[0,1\right]\right)\times vec_{i}^{g}$$ (14)

4) *Global optimum scheme*: Based on the newly proposed *global optimum scheme*, the mutation factor *F* and crossover factor *CR* were updated. The *global optimum scheme* is used in two scenarios: 1) when *f(nvecig)* is smaller than or equal to *f(α*_*base*_^*g+1*^), we let *nvec*_*i*_^*g*^ equal *α*_*i*_^*g*^, and 2) when *f(vecig)* equals *f(α*_*i*_^*g*^ *)*, the previous individual *q*_*i*_^*g*^ is set to the next individual *q*_*i*_^*g+1*^.

##### 2.2.2.2 Pseudo-code of our MQDE method

We used a memory-based scheme to save the optimal mutation factor *F* and the crossover rate *CR* from the former iteration, and then used them as the initialization for the next iteration. Based on this scheme, we were able to find the optimal candidate. The pseudo-code of our MQDE method is shown in Algorithm 1.

Algorithm 1 MQDE algorithm.

01:Generate a uniformly distributed random initial population containing *NP* solutions, which include *NI* variables according to *α*_*i*_^*0*^ = *α* ^*min*^ + *rand*[0, 1] * (*α* ^*max*^ *- α* ^*min*^) (*i*∈[1, *NI*]). Due to its DE/rand/1 scheme (shown in Eq. 26), *NI* is equal to 3. We initialized the current iteration number *g* = 1, defined *g* < *g*_max_ (maximum iteration number), and set the initial *F* and CR as ∈(0, 1]. The population size *NP* was obtained according to *NP* = (*I* +1) *H + (*H* + 1) *K, where *I* denotes the number of input neurons in the NNs, *H* is the number of hidden neurons in the NN, and *K* is the number of output neurons in the NN.
02:while *g* < *g*_max_
03:for *i* = 1 to *NP*//*memory-based mechanism*
04:Generate three random indices *r*_*1*_, *r*_*2*_, and *r*_*3*_ with *r*_*1*_ ≠ *r*_*2*_ ≠ *r*_*3*_ ≠ *i*//*new quantum mutation*
05:*λ =* ((*g*_max_ *- g*)*/g*_max_)^*2*^
06:*α*_*superior*_^*g*^ *= math.floor* (*α*_*i*_^*g*^ * NI)
07:*α*_*base*_^*g*^ = λ * α_*i1*_^*g*^ *+* (*1-λ*) *α_*superior*_^*g*^
08:*vec*_*i*_^*g*^ *= α*_*i1*_^*g*^ *+ F* * (*α*_*i2*_^*g*^ *-α*_*i3*_^*g*^ *)*//* end new quantum mutation*
09:if *i* > 2 //*CS method*
10:*Levy*(*ap*) = *g* ^*-ap*^
11:*α*_*base*_^*g*^ *= α*_*base*_^*g-1*^ *+Levy* (*ap*)
12:*nvec*_*i*_^*g*^ = *rand*[0, 1] *α_*base*_^*g*^ *+* (*1- rand[0, 1]*) * *vec*_*i*_^*g*^
13:else
14:*nvec*_*i*_^*g*^ = *rand*[0, 1] *α_*base*_^*g*^ *+* (*1- rand[0, 1]*) * *vec*_*i*_^*g*^ // *end CS method*
15:if rand[0, 1] ≤ *CR *// *quantum crossover*
16:*u*_*i*_^*g+1*^ = *nvec*_*i*_^*g*^
17:else
18:u_i_^g*+1*^ = *α*_*base*_^*g*^
19:end if // *end quantum crossover*
20:if *f*(*u*_*i*_^*g*^) ≤ *f*(*α*_*base*_^*g*^) // *quantum selection*
21:*α*_*base*_^*g+1*^ *= u*_*i*_^*g*^
22:else
23:*α*_*base*_^*g+1*^ = *α*_*base*_^*g*^
24:end if
25:if *f*(*u*_*i*_^*g+1*^) ≤ *f*(*α*_*base*_^*g*^)
26:*q*_*i*_^*g+1*^ = *u*_*i*_^*g+1*^
27:else
28:*q*_*i*_^*g+1*^ = *α*_*base*_^*g+1*^
29:end if // *end quantum selection*
30:if *f*(*vec*_*i*_^*g*^) = = *f*(*α*_*i*_^*g*^) // *global optimum scheme*
31:*q*_*i*_^*g+1*^ = *q*_*i*_^*g*^
32:if *f*(*nvec*_*i*_^*g*^) ≤ *f*(*α*_*base*_^*g+1*^)
33:*α*_*i*_^*g*^ = *nvec*_*i*_^*g*^ // *end global optimum scheme*
34:Update *F* and *CR* according to Eqs. 15–18
35:end for
36:*g* = *g* + 1 // *end memory-based mechanism*
37:end while

The workflow of the MQDE method is shown below:

Step 1: Initialize the MQDE method.

Step 2: Obtain the newly generated mutant individual *nvec*_*i*_^*g+1*^ based on the *new quantum mutation technique* and the *CS method* in the quantum mutation step, as shown in Eqs. 9–14.

Step 3: Based on Eq. 30, the experimental individual *u*_*i*_^*g+1*^ is achieved in quantum crossover step.

Step 4: Using Eqs. 31, 32, update *α*_*base*_^*g+1*^ and *q*_*i*_^*g+1*^ in the quantum selection step.

Step 5: The *global optimum scheme* should be met, as shown in Section 2.2.2.1.

Step 6: During the updating process, renew both *F* and *CR* according to Eqs. 15, 16.

$$F=\left(1-val\right)\times F+rand\left[0,1\right]\times mean_{L}\left(S_{F}\right)$$ (15)

and

$$CR=\left(1-val\right)\times CR+rand\left[0,1\right]\times mean_{L}\left(S_{CR}\right)$$ (16)

where *S*_*F*_ and *S*_*CR*_ represent the successful mutation and crossover probabilities, respectively. The adjustment parameter *val* is randomly selected within (0, 1]. The Lehmer mean *mean*_*L*_(*•*) (Cui et al., 2018) is applied to renew the values of *F* and *CR* according to Eq. 17 and Eq. 18.

$$mean_{L}\left(S_{F}\right)=\frac{\sum_{F\in S_{F}}F^{2}}{\sum_{F\in S_{F}}F}$$ (17)

$$mean_{L}\left(S_{CR}\right)=\frac{\sum_{CR\in S_{CR}}CR^{2}}{\sum_{CR\in S_{CR}}CR}$$ (18)

Step 7 Update both *F* and *CR* based on the *storage-based mechanism* (Chen et al., 2020).

When *g* < *g*_*max*_, and *g* = *g* + 1, then step (2) is executed, in which the optimal *uF* and *uCR* in the current iteration are used for the next iteration; if *g* ≥ *g*_*max*_, it proceeds to the next step.

Step 8 Determine the optimal individual.

#### 2.2.3 Stage 3: training

In the backpropagation neural network (BPNN), the gradient descent method is often used to decrease the deviation between the actual and desired outputs (Peng et al., 2022b). We used the FBNNL (Chen et al., 2020), which inherits the storage and heredity abilities of the Caputo-type fractional gradient descent method (Xiao et al., 2015) but also inherits the ability to combat overfitting without revising the network architecture from *L*_*2*_ regularization. As a three-layer NN is able to approximate various nonlinear functions with any expected precision (Peng et al., 2022a), we used a three-layer FBNNL. Furthermore, the sigmoid and ELU functions (Peng et al., 2022c) were used in the forward propagation step. Two units were included in the output layer, i.e., *Output*(*x*) and *Output*(*y*), which are regarded as the expression functions *Output*(*x*(*t*)) and *Output*(*y*(*t*)), respectively, on the projection index *t*.

#### 2.2.4 Stage 4: interpretable model-based contour extraction

After obtaining the optimal FBNNL, we first developed a smooth and interpretable mathematical definition of the organ contour, which is denoted by the parameters of FBNNL, as shown below:

$$f\left(t\right)=\left(x\left(t\right)\right),\left.y\left(t\right)\right)=\left(\frac{2\times Output\left(x\left(t\right)\right)+1}{2\times Output\left(x\left(t\right)\right)+2},\frac{2\times Output\left(y\left(t\right)\right)+1}{2\times Output\left(y\left(t\right)\right)+2}\right)$$ (19)

where *x*(*t*) and *y*(*t*) were used to show the x-axis and y-axis coordinates of the points of the resulting contour, respectively. *Output*(*x*(*t*)) and *Output*(*y*(*t*)) are shown as below:

$$\left(Output\left(x\left(t\right)\right),Output\left(y\left(t\right)\right)\right)=\left(\frac{e^{\sum_{j=1}^{K}\frac{1}{1+e^{\sum_{i=1}^{H}-\left(tw1_{i}-a_{i}\right)}}w_{2j,1}-b_{j,1}}-1}{2},\frac{e^{\sum_{j=1}^{K}\frac{1}{1+e^{\sum_{i=1}^{H}-\left(tw1_{i}-a_{i}\right)}}{w_{2j}}_{,2}-b_{j,2}}-1}{2}\right)$$ (20)

where *K* and *H* denotes the number of output and hidden neurons, respectively; *b*_*j*_ (*j = 1, 2*) is the output threshold of the *j-th* neuron at the output layer; and *w*_*1*_ and *w*_*2*_ are the hidden and output weights, respectively. In addition, for better understanding, we have introduced the architecture of BPNN and FBNNL in Supplementary Appendix S3, S4, respectively. Meanwhile, the procedure of achieving Eqs. 19, 20 are shown in Supplementary Appendix S5.

### 2.3 Materials

Two datasets, namely, a transrectal ultrasound prostate set and a trans-abdominal ultrasound kidney set, were used in our experiments. Here, we mainly illustrate the details of these datasets.

#### 2.3.1 Jiangsu province hospital of chinese medicine prostate dataset (JPHCM)

This prostate dataset contains 393 slices. All of the prostate images were obtained using the ultrasound imaging workstation VINNO 70LAB and an ultrasound probe with a frequency of 4–8 MHz. The size of each slice was 1,200 × 900 pixels.

#### 2.3.2 Suzhou municipal hospital kidney dataset (SMH)

This kidney dataset was collected using the Mindray DC-8 diagnostic ultrasound system (Mindray Medical International Limited, Shenzhen, China), with an integrated low-resolution linear transducer with a frequency of 1.3–5.7 MHz. The device parameters comprised a mechanical index of 1.3, a probing depth of 200 mm, and an amplifier gain within 3–33 dB. The resolution of this SMH set was also 1,200 × 900 pixels.

Table 1 shows the distribution of the images from both datasets. The two datasets (i.e., JPHCM and SMH) were used to generate a new dataset called the *combined dataset* for evaluation. Due to the limited amount of training data in the JPHCM dataset, we randomly rotated the training data within [-15°, 15°], where each original image was rotated three times. All of the ultrasound slices were resampled to a unified resolution of 600 × 450 pixels. Based on our previous research (Peng et al., 2018b), we set 10 hidden neurons and 1,000 epochs for the FBNNL to simplify the complexity of the NN model and prevent overfitting. We used the DSC, Jaccard similarity coefficient (OMG), and accuracy (ACC) (Peng et al., 2020) as the evaluation metrics. All of the ground truths were labeled and verified by three physicians. The algorithm ran on a Windows 10 desktop with an Intel Core i7-8750H CPU (3.9 GHz with six cores) and a GTX 1070 with Max-Q design GPU.

**TABLE 1:** The distribution of images of both datasets.

	Total set	Training set (raw + augmentation)	Validation set	Testing set
JPHCM	393	215 (raw) + 645 (aug)	70	108
SMH	1380	960 (raw)	144	276
*Combined dataset*	-	1820	214	384

## 3 Results

We first assessed the capability of our method on multiple datasets (Section 3.1), and then assessed the robustness of our method on a testing set with various degrees of corruption (Section 3.2). Next, we determined the influence of each component of our method using an ablation experiment (Section 3.3). Finally, we compared our method with current state-of-the-art algorithms (Section 3.4).

### 3.1 Model selection

#### 3.1.1 Evaluation of our method with and without preprocessing

As shown in Table 2, three metrics (i.e., DSC, OMG, and ACC) were used to investigate whether using a preprocessing stage affected the testing performance of our method. We used the ELU activation function, one hidden layer, and stochastic gradient descent (SGD). From the data presented in Table 2, we can see that the use of a preprocessing stage consisting of Z-score normalization and histogram equalization schemes improved the performance (i.e., precision and robustness) of our method (*Preprocess*). For the following experiments, the preprocessing stage was included in all of the methods.

**TABLE 2:** Evaluation of our method with and without preprocessing process.

Method	DSC (%)	OMG (%)	ACC (%)
Our method (*not Preprocess*)	95.6 ± 2.5	94.5 ± 2.7	95.3 ± 2.6
Our method (*Preprocess*)	95.7 ± 2.4	94.6 ± 2.6	95.3 ± 2.6

#### 3.1.2 Training process on various optimizers

**FIGURE 5:**
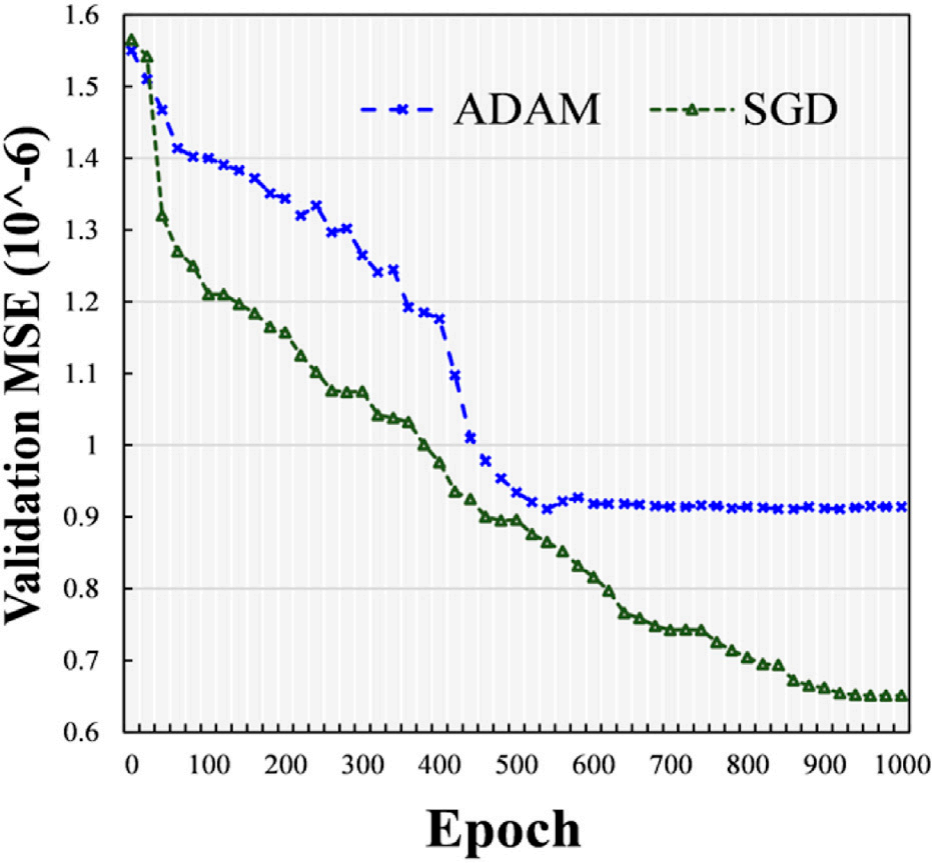
Comparison of validation mean square errors for various optimizers.

Here, we investigated the influence of different optimizers, such as the SGD technique (Amari, 1993) and Adam optimizer (ADAM) (Bock and Weis, 2019), and we used one hidden layer and the ELU activation function. The experimental outcomes in terms of the mean square error (MSE) with different epochs using various optimizers (i.e., SGD and ADAM) were determined on the validation dataset. At various epochs, the validation MSE values of various optimizers (i.e., SGD and ADAM) are indicated in Figure 5. From the data presented in Figure 5, it can be seen that during the training stage, the ADAM optimizer approached stability at approximately 500 epochs, while the SGD continued training until 1,000 epochs. Compared with the model using ADAM, the one using SGD obtained a lower validation MSE with more accurate results. Therefore, we used the SGD technique as the optimizer.

#### 3.1.3 Selection of the optimal number of hidden layers

For the neural network (the NN), the option of the number of hidden layers has a significant influence on the training accuracy and computational efficiency. Table 3 presents the evaluation at different hidden layers, with the SGD and ELU activation functions adopted for our model. From 1 to 3 hidden layers, the DSC slightly increased by 0.2%, but the testing time was more than twice as long. When the number of hidden layers continued to grow, the DSC did not continue to increase but decreased. Meanwhile, the testing time increased. The possible cause of this phenomenon is that more hidden layers make the network more complex, which leads to an increase in the computational efficiency of the network and the appearance of overfitting. Overall, we used the NN containing one hidden layer.

**TABLE 3:** Evaluation of different hidden layers.

Layers	DSC (%)	OMG (%)	ACC (%)	Testing time (s)
1	95.7 ± 2.4	94.6 ± 2.6	95.3 ± 2.6	7
2	95.7 ± 2.4	94.7 ± 2.5	95.3 ± 2.6	10
3	95.9 ± 2.4	94.9 ± 2.5	95.4 ± 2.5	15
4	95.4 ± 2.7	94.3 ± 2.7	95.1 ± 2.8	22
5	95 ± 3.1	93.8 ± 3.2	94.7 ± 3.1	31

#### 3.1.4 Selection of the optimal activation function

In this subsection, we assessed the effect of various activation functions, including Tanh, ReLU, and ELU, while using one hidden layer and an SGD optimizer. Table 4 presents the evaluation of different activation functions. As shown in Table 4, our method using ReLU and ELU activation functions performed better than the method using the Tanh function model. The main reason for this phenomenon is that both activation functions were able to solve the gradient vanishing issue. Our method using the ELU function had more satisfactory performance than the method using the ReLU function. Hence, we used the ELU function for subsequent experiments.

**TABLE 4:** Evaluation between our method with and without preprocessing process.

Activation function	DSC (%)	OMG (%)	ACC (%)
Tanh	95.1 ± 2.8	93.7 ± 3.2	94.6 ± 3.1
ReLU	95.4 ± 2.5	94.3 ± 2.9	95.2 ± 2.6
ELU	95.7 ± 2.4	94.6 ± 2.6	95.3 ± 2.6

### 3.2 Segmentation performance on multiple datasets

**FIGURE 6:**
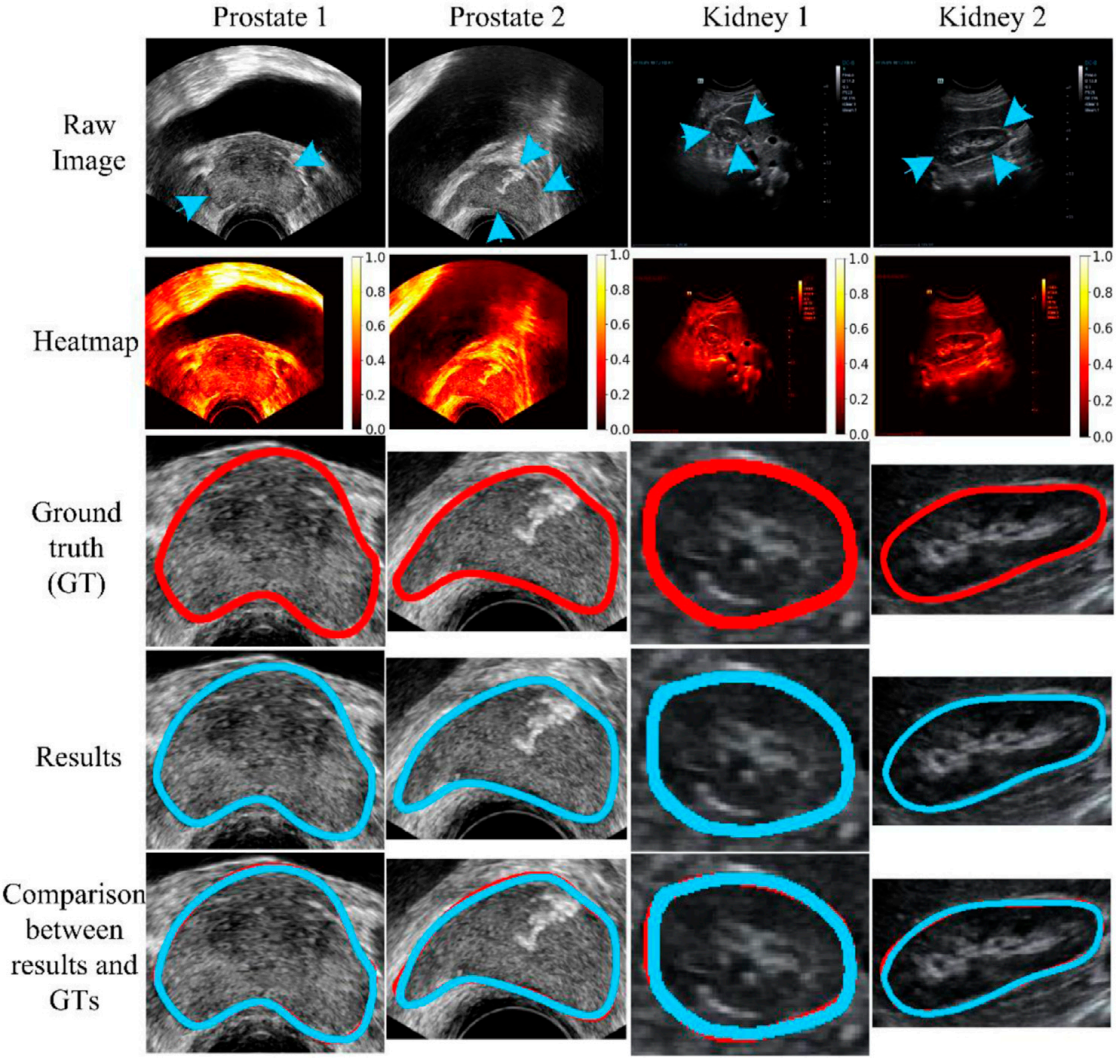
Qualitative evaluation. The blue arrow indicates the missing or unclear boundaries of the organs. The blue arrow indicates the weak edge region (i.e., missing or unclear boundaries) of the organs. The weak edge region in the prostate image (left two images) is caused by the surrounding tissues and involves intestinal gas. In addition, in the kidney image (right two images), the front weak edge region is caused by the influence of the liver, and other weak edge regions are caused by intestinal gas. The blue and red curves represent the experimental results and ground truth (GT), respectively.

Figure 6 shows four ultrasound results (Image 1-Image 4) randomly selected from the total testing outcomes for qualitative evaluation. The first two columns show randomly chosen results from the prostate dataset (JPHCM dataset), and the second two columns show results stochastically selected from the kidney dataset (SMH dataset). The first three rows present the raw data, its corresponding heatmap, and ground truth (GT), respectively. The last two rows show the experimental results and compared results, respectively. The “compared results” in the fifth row represent the comparison between the segmentation result and the GT. It can be seen from Figure 6 that the experimental outcomes acquired satisfactory similarities with the GTs.

### 3.3 Evaluation of robustness of our model

**FIGURE 7:**
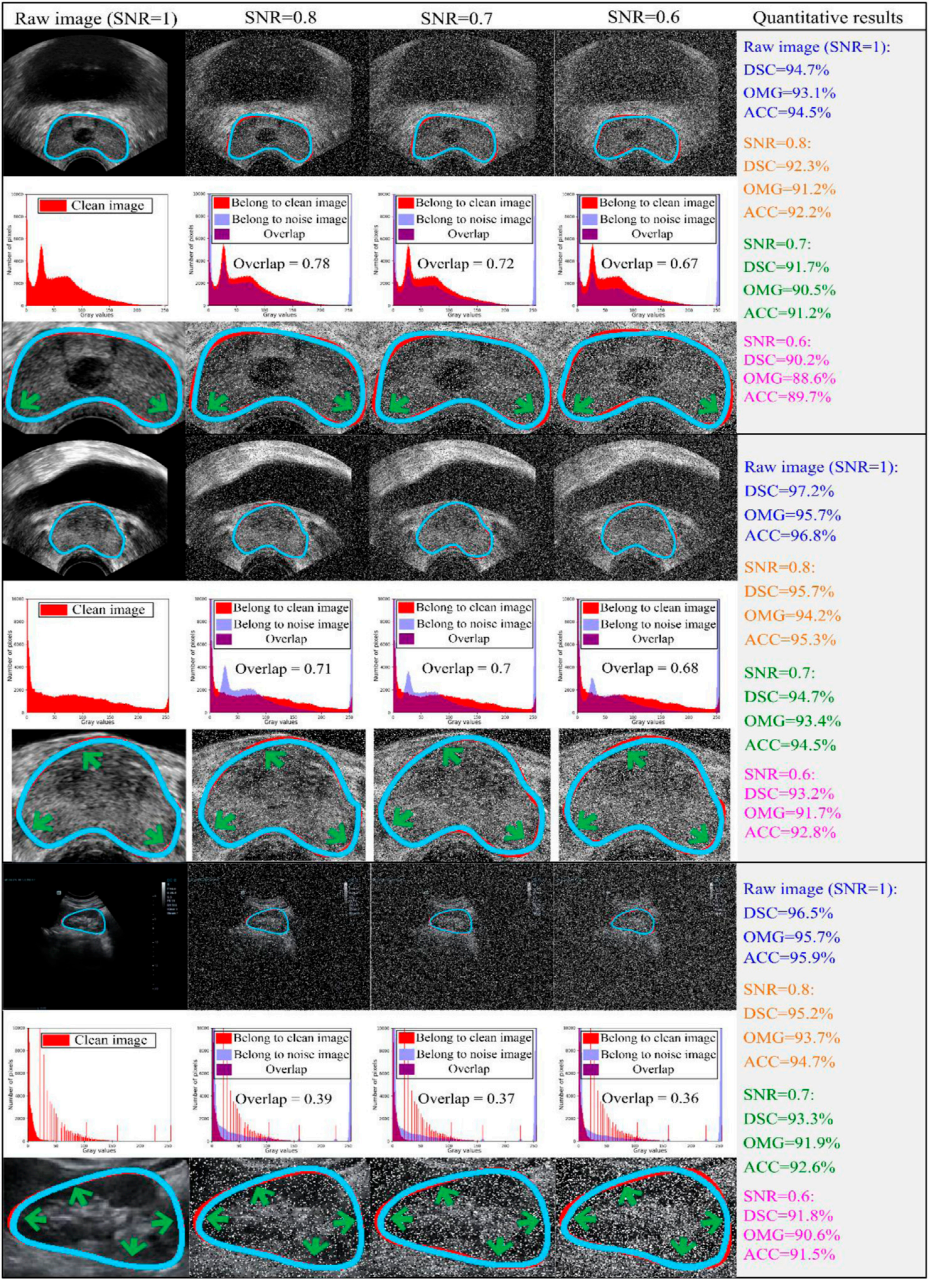
Three cases are randomly chosen from both datasets for evaluation. The first two-three rows indicate the experimental results from JPHCM prostate data, and the last three rows represent the experimental results from the SMH kidney dataset. The first, fourth, and seventh rows show the comparison between the experimental result and ground truth, where the blue and red curves show the experimental result and ground truth, respectively. The second, fifth, and eighth rows indicate the histogram overlap between the clean image and noise image. The last three rows (i.e., third, sixth, and ninth rows) show the zooming display of the region of interest. The experimental outcomes are shown at various signal-to-noise ratios (SNRs; i.e., 0.6, 0.7, and 0.8). Thus, testing images damaged by different levels of noise were used to evaluate the capability of our method. The experimental outcome at an SNR of 1 demonstrated that our method was assessed on raw/clean testing data. Here are the abbreviations used in the figure: SNR: signal-to-noise ratio; DSC: Dice similarity coefficient; SD: standard deviation; OMG: Jaccard similarity coefficient; ACC: Accuracy.

To evaluate the robustness of our model, we used different signal-to-noise ratios (SNRs) of salt and pepper noise to corrupt the testing image set, and the damaged testing images were then used to assess the capability of our model. We set the values of SNR to 1, 0.8, 0.7, and 0.6. The testing outcomes of our method are described in Table 5, while Figure 7 shows the qualitative outcomes of our method in three randomly selected cases. Meanwhile, the overlapped region (*overlap*) (Ali and Madabhushi, 2012) was used as another evaluation metric, calculated as:

$$overlap=\frac{\left|cleanG\cap noiseG\right|}{cleanG}$$ (21)

where *overlap* indicates the proportion of overlap between the gray values of the clean image (*cleanG*) and the noisy image (*noiseG*).

**TABLE 5:** Results using different SNRs of the salt and pepper noise. We use the format with “mean value ± standard deviation (%)” to denote each evaluation metric. In addition, the results on different SNRs (i.e., 0.6, 0.7, and 0.8) indicate that our method used images corrupted by different levels of noise for testing. However, the result on SNR = 1 shows that our method was evaluated on raw/clean data.

	DSC ±SD (%)	OMG ±SD (%)	ACC ±SD (%)
Clean data (SNR = 1)	95.7 ± 2.4	94.6 ± 2.6	95.3 ± 2.6
SNR = 0.8	94.6 ± 2.7	93.1 ± 3.3	94.2 ± 2.8
SNR = 0.7	93.5 ± 3.2	92.2 ± 3.7	93.2 ± 3.5
SNR = 0.6	91.6 ± 4.4	90.7 ± 4.5	91.2 ± 4.4

SNR, signal-to-noise ratio; DSC, dice similarity coefficient; SD, standard deviation; OMG, jaccard similarity coefficient; ACC, accuracy.

As shown in Table 5, as the SNR was reduced from 1 to 0.6, the mean values of DSC, OMG, and ACC decreased by 4.47%, 4.29%, and 4.49%, respectively. When the SNR equaled 0.6, our model had the lowest performance, with DSC = 91.6 ± 4.4 (%), OMG = 90.7 ± 4.5 (%), and ACC = 91.2 ± 4.4 (%). Figure 7 shows that our method achieved a similar performance in both the prostate and kidney datasets. Therefore, we mainly discuss the performance of our method in the prostate dataset. As the SNR reduced from 0.8 to 0.6, the *overlap* rate reduced from 0.78 to 0.67, while the mean values of DSC, OMG, and ACC decreased by 2.32%, 2.93%, and 2.78%, respectively.

Overall, the mean values of all the metrics, including the DSC, OMG, and ACC, were greater than 90.5%, which further demonstrated that our method was able to handle noisy data well.

### 3.4 Ablation study

In this section, we report the quantitative and qualitative evaluation of the performance of our method using an ablation study (AS). The results of the AS are shown in Table 6. The AS was mainly used to evaluate whether the capability of our method was affected when several components of the method were replaced or removed (Cashman et al., 2019). The principal components of our approach contained MS-based, DE-based, and NN-based modules. As shown in Table 6, using AS1 as the baseline achieved the lowest DSC, OMG, and ACC values of 91.6% ± 4.3%, 90.2% ± 5%, and 91.2% ± 4.4%, respectively. Based on AS1, we used another component (i.e., NSMS, MQDE, or FBNNL), and the mean DSC, OMG, and ACC values increased by 1.63%-4.47%, 1.33%-4.87%, and 1.53%-4.49%, respectively.

**TABLE 6:** Ablation results. The description format of each result is “mean value ± standard deviation (%)”. All the methods have contained the preprocessing stage.

	Model	Results
AS1	MSC + projection + QDE + BPNN (baseline)	DSC = 91.6 ± 4.3
		OMG = 90.2 ± 5
		ACC = 91.2 ± 4.4
AS2	NSMS + projection + QDE + BPNN	DSC = 93.1 ± 3.5
		OMG = 91.4 ± 4.5
		ACC = 92.6 ± 3.8
AS3	NSMS + projection + MQDE + BPNN	DSC = 94.8 ± 2.6
		OMG = 93.5 ± 3.2
		ACC = 94.2 ± 2.9
Our method (AS4)	NSMS + projection + MQDE + FBNNL	DSC = 95.7 ± 2.4
		OMG = 94.6 ± 2.6
		ACC = 95.3 ± 2.6

AS, ablation study; MSC, mean shift clustering; NSMS, neutrosophic-set-based mean shift method; QDE, Quantum-inspired differential evolution; MQDE, memory-based quantum-inspired differential evolution; BPNN, backpropagation neural network; FBNNL, Fractional-order backpropagation neural network; DSC, dice similarity coefficient; SD, standard deviation; OMG, jaccard similarity coefficient; ACC, accuracy.

**FIGURE 8:**
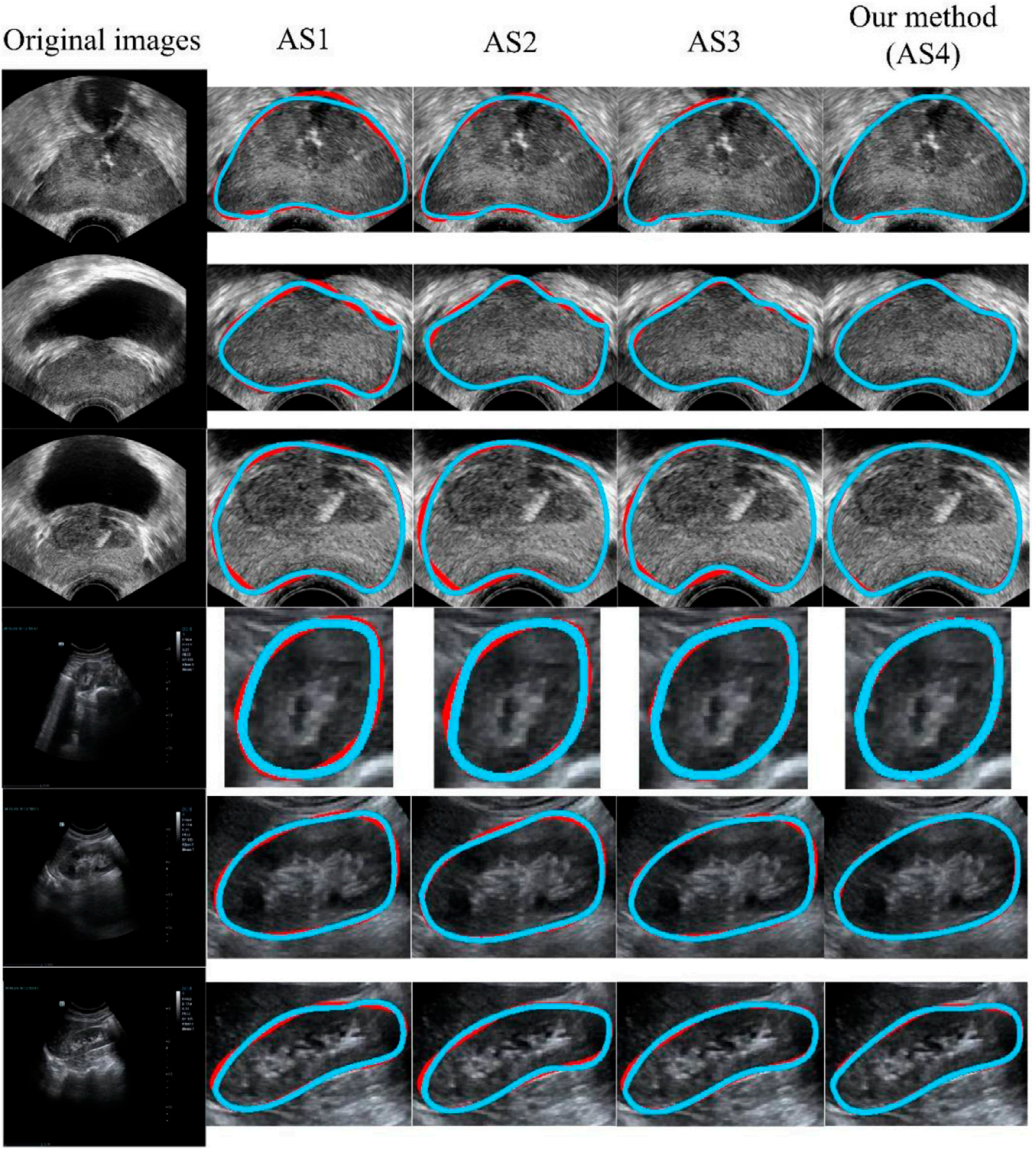
Qualitative results using different ASs. The blue and red curves show the experimental results and ground truth, respectively. From AS1 to AS4, the performance increased progressively. AS4 represents our method. All of the methods (AS1-AS4) included the preprocessing stage. AS: ablation study.

Our model (AS4) had the optimal results, with DSC, OMG, and ACC values of 95.7% ± 2.4%, 94.6% ± 2.6%, and 95.3% ± 2.6%, respectively. Figure 8 presents a visual comparison of three randomly selected segmentation outcomes. The first three rows show the results of randomly chosen prostate cases, and the last three rows show the results of kidney cases. Table 7 represents the corresponding quantitative outcome of each qualitative outcome in Figure 8, where various evaluation metrics, including DSC, OMG, and ACC are adopted for assessment.

**TABLE 7:** Corresponding quantitative result of each qualitative result in Figure 8, where different metrics (i.e., DSC, OMG, and ACC) are used for evaluation.

AS1	AS2	AS3	Our method (AS4)
DSC = 89.3% OMG = 87.6% ACC = 88.8%	DSC = 91.7% OMG = 90.3% ACC = 91.2%	DSC = 94.1% OMG = 92.8% ACC = 94%	DSC = 97% OMG = 95.9% ACC = 96.7%
DSC = 91.3% OMG = 90.2% ACC = 91%	DSC = 93.3% OMG = 91.6% ACC = 92.9%	DSC = 94.6% OMG = 93.3% ACC = 93.9%	DSC = 97.1% OMG = 95.9% ACC = 96.8%
DSC = 92.8% OMG = 91.6% ACC = 92.3%	DSC = 93.2% OMG = 91.6% ACC = 92.6%	DSC = 93.4% OMG = 92.1% ACC = 93.2%	DSC = 97.1% OMG = 95.4% ACC = 96.6%
DSC = 90.9% OMG = 89.9% ACC = 90.6%	DSC = 94.1% OMG = 92.8% ACC = 93.5%	DSC = 95.9% OMG = 94.4% ACC = 95.6%	DSC = 97.8% OMG = 96.6% ACC = 97.3%
DSC = 93.3% OMG = 91.9% ACC = 92.6%	DSC = 94.9% OMG = 93.8% ACC = 94.3%	DSC = 95.7% OMG = 94.2% ACC = 95.5%	DSC = 97.6% OMG = 96.6% ACC = 97.3%
DSC = 92.4% OMG = 90.7% ACC = 92%	DSC = 93.8% OMG = 92.2% ACC = 93.1%	DSC = 94.1% OMG = 92.9% ACC = 93.8%	DSC = 95.9% OMG = 94.3% ACC = 95.4%

### 3.5 Comparison with state-of-the-art methods

Table 8 shows the quantitative outcomes obtained after comparisons with multiple state-of-the-art methods: Hull-CPL (Peng et al., 2019), H-SegMed (Peng et al., 2022a), Mask-RCNN (He et al., 2017), Unet++ (Zhou et al., 2020), UTNet (Gao et al., 2021), and UNETR (Hatamizadeh et al., 2022). These methods are grouped into two categories: hybrid methods (Hull-CPL (Peng et al., 2019) and H-SegMed (Peng et al., 2022a)) and deep-learning methods (Mask-RCNN (He et al., 2017); Unet++ (Zhou et al., 2020); and two Transformer-based architectures, UTNet (Gao et al., 2021) and UNETR (Hatamizadeh et al., 2022)).

**TABLE 8:** Results of all the methods.

References	Method	Model	DSC (%)	OMG (%)	ACC (%)
He et al. (2017)	Mask-RCNN	Deep learning	90.4 ± 6.3	89.4 ± 6.1	90.2 ± 6.4
Zhou et al. (2020)	Unet++	Deep learning	90.7 ± 5.7	89.1 ± 6.5	90.1 ± 5.8
Gao et al. (2021)	Transformer-based UTNet	Deep learning	91 ± 5.2	89.7 ± 6.4	90.4 ± 5.4
Hatamizadeh et al. (2022)	Transformer-based UNETR	Deep learning	91.1 ± 5.2	90.1 ± 6.1	90.8 ± 5.3
Peng et al. (2019)	Hull-CPL	Hybrid	94.1 ± 2.9	92.7 ± 3.3	93.7 ± 3
Peng et al. (2022a)	H-SegMed (IJCV-2022)	Hybrid	95.2 ± 2.5	94.1 ± 2.8	95 ± 2.7
Our method	-	Hybrid	95.7 ± 2.4	94.6 ± 2.6	95.3 ± 2.6

DSC, dice similarity coefficient; SD, standard deviation; OMG, jaccard similarity coefficient; ACC, accuracy; CPL, closed polygonal line method; Mask-RCNN, mask region-based convolutional neural network method.

In this comparison, the hybrid methods are three-layer-based frameworks, where the sigmoid and ELU functions are used in hidden and output layers, respectively. Meanwhile, the methods used the SGD scheme (Qian et al., 2015) as an optimizer, in which the initial learning rate, momentum value, and the maximum number of epochs were 0.4, 0.9, and 1,000, respectively. In addition, all of the deep-learning methods used the Dice loss function during training, where the initial value of the learning rate was set to 10e-3 and reduced to a plateau with patience of 50 and a maximum number of epochs of 1,000. All of the models used the same training, validation, and testing datasets. The proportions of the datasets used are described in Section 2.3.

As shown in Table 8, the hybrid models differed from the deep-learning models, as they had more correct segmentation outcomes with less training data. This illustrates that the combination of PC-based and NN-based models is good at data fitting. Overall, our proposed method is promising.

## 4 Conclusions and discussion

Due to blurry boundaries and the existence of shadow artifacts in ultrasound images, accurate ultrasound organ segmentation is challenging. We developed a hybrid segmentation network for ultrasound images. Differing from previously reported models, our model has four main metrics and contributions. First, current medical segmentation models are principally classified into two groups: fully automatic and semi-automatic models. Due to the challenges associated with ultrasound organ segmentation, the mean DSC of fully automatic methods is approximately 0.9 (Girum et al., 2020; Wang et al., 2019), while our proposed framework achieved a mean DSC as high as 0.957 (shown in Table 8). Therefore, the number of images in the training datasets for several deep-learning-based segmentation methods is more than 4,000 slices, with a DSC of 0.92 (Lei et al., 2019), but we used fewer images for training and achieved a higher DSC. The primary reason is that our method introduced the characteristics of the PC by fitting the center of the dataset automatically while using only a few points as the prior. Second, standard PC-based methods cannot determine the number of cluster vertices automatically; rather, it is pre-decided by the users. Hence, these methods achieve variable outcomes based on different pre-set numbers of cluster vertices (shown in Figure 1). Due to this issue, we used the ASPC model to decide the number of cluster vertices automatically without prior knowledge. Third, we used modified quantum-inspired differential evolution to assist FBNNL to find the optimal model so that we could avoid FBNNL trapping into the local optimum during training. Fourth, considering that the outcomes of standard PC-based methods are not smooth (shown in Figure 1), we designed an explicable mathematical formula to smooth the organ contour, which is expressed by the parameters of the FBNNL. In this section, we analyze the entire study from various views.

### 4.1 Effect of noise (noise level)

To determine the robustness of our method, we used corrupted testing data for evaluation, as described in Section 3.2. Over the past several years, the influence of SNR on salt and pepper noise has been discussed after multiple trials. For example, Tang *et al*. (Tang et al., 2020) added salt and pepper noise to their testing data to evaluate the robustness of their method, producing SNRs in the range of [0.8, 0.95]. In addition, Benaichouche et al. (Benaichouche et al., 2013) designed an improved fuzzy clustering segmentation algorithm, including medical brain images, where the SNR was set to 0.9. A smaller SNR is known to cause more damage to the image (Tang et al., 2020). In our study, a higher level of damage to the testing images was used, with SNRs set as low as 0.6, making it more difficult to achieve a precise result. Nevertheless, our method achieved excellent results (all metrics > 90.5%, Table 5) with various degrees of salt and pepper noise, illustrating the robustness of our model.

### 4.2 Degree of damage to the image by noise (histogram)

Ultrasound images are gray-scale images, in which the primary region is the black pixel region (gray value = 0). This makes it challenging to separate the ROI in ultrasound images according to black pixels. Considering the distribution of pixels in ultrasound data, we chose the number of pixels within the range [0, 10,000]. As the SNR reduced from 1 to 0.6, an increasing amount of white noise was added, resulting in fewer raw image pixels. In other words, the raw image was seriously damaged by white noise (gray label = 255). Overall, the mean values of all the metrics were greater than 90.5% (shown in Table 5), demonstrating that even the outlines with vague regions were detected accurately.

### 4.3 Large amount of bias between prostate and kidney histograms

As shown in Figure 7, there was a large amount of bias when comparing the histogram overlap of the transrectal prostate image (second and fifth rows in Figure 7) and the trans-abdominal kidney image (eighth row in Figure 7). Although damaged by the same degree of salt and pepper noise, the value of histogram overlap of the prostate was close to double that of the kidney. The primary cause of this result is that, due to the short penetration distance between the rectum and the prostate, the process of detection of a transrectal prostate image can be completed quickly. Therefore, the detection process may be less influenced by neighboring tissues, and the pixel value in the ultrasound image changes steadily. Nevertheless, during the detection of trans-abdominal kidney images, the detection probe is inserted through the abdomen by radiologists. As the abdominal cavity is a hollow organ, it causes a long penetration distance. When imaging the kidney, the serious attenuation of ultrasonic waves is affected by the long penetration distance, and the mutual influence of different organs is larger.

### 4.4 Degree of difficulty degree with multi-organ ultrasound tasks

As presented in Section 3.4, both hybrid models (our current model and our previous H-SegMed model) obtained excellent segmentation results. Nevertheless, compared with the current model, the former H-SegMed model (Peng et al., 2022a) had 0.52%, 0.53%, and 0.31% lower DSC, OMG, and ACC values, respectively. Using a larger training dataset increased the robustness of the H-SegMed model. Meanwhile, there were two reasons for the reduced accuracy of the H-SegMed model. First, in the recent task, the H-SegMed model used various datasets with different organs for training, which increased the difficulty of the segmentation task. Second, a larger image resolution was used, which increased the difficulty of the segmentation task (Candemir et al., 2014).

Our method obtained excellent results, but some aspects could be optimized to further improve its capability. *First*, there are three cascaded stages in our method, which increases the memory burden during the segmentation task. Hence, squeezing the memory of the model needs to be considered in the future. *Second*, the robustness and accuracy of our method could be evaluated in different situations. For example, unusual or changeable motion of the organs (i.e., prostate and kidney) would influence our model’s performance. Moreover, the precision of our model may be significantly altered for different age groups or sexes. *Third*, in the future, the capability of our method will be further evaluated for various imaging modalities such as computed tomography and magnetic resonance imaging. *Fourth*, we aim to convert the semi-automatic model to a fully automatic model, which will be more suitable for real-time clinical applications.

## Data Availability

The original contributions presented in the study are included in the article/Supplementary Material, further inquiries can be directed to the corresponding authors.
