# Supplementary material for: Intelligent contour extraction approach for accurate segmentation of medical ultrasound images
Source: Front Physiol. 2023 Aug 22;14:1177351. doi: 10.3389/fphys.2023.1177351 (PMC10479019; doi:10.3389/fphys.2023.1177351)
Supplement: Supplementary file 1 [file DataSheet1.docx]

# Appendix

## Quantum characteristics

For the quantum-inspired differential evolution (QDE) method [51] [52], quantum computing was introduced into the traditional differential evolution (DE) method and combined with quantum characteristics to enhance the optimization capability of the DE method. The main concepts of the quantum characteristics are described below.

*1) Real number-based chromosome coding:* the quantum bit is regarded as the smallest unit of information and is deposited in the quantum computer with “0” and “1” states. We suppose α and β to represent the probability amplitudes of the “0” and “1” states, respectively, where both α and β are complex numbers. At the same time, the probability of calculating |0〉 and |1〉 represent |α|^2^ and |β|^2^, respectively. The quantum bit is represented by,

$|\psi\rangle=\alpha|0\rangle+\beta|1\rangle$ (22)

where it satisfies |α|^2^ + |β|^2^ = 1.

*2) Q-bit representation:* The Q-bit representation is mainly used to indicate a linear superposition of states. It consists of a string of m Q-bits, and the Q-bit individual is represented by:

$q=\left[\left.\begin{matrix} \alpha_{1} \\ \beta_{1} \end{matrix}\right|\left.\begin{matrix} \alpha_{2} \\ \beta_{2} \end{matrix}\right|\left.\begin{matrix} \cdots\\ \cdots\end{matrix}\right|\begin{matrix} \alpha_{m} \\ \beta_{m} \end{matrix}\right]$ (23)

*3) Quantum rotation gate:* The quantum rotation gate is significant for updating the relative phase among quanta to enhance the performance of both global and local searches of the QEN method. Based on a rotation angle $\Delta\theta_{i},i=1,...,m$, the quantum rotation gate $R(\theta_{i})$ is described by:

$R(\theta_{i})=\left[\begin{matrix} \cos(\Delta\theta_{i}) & -\sin(\Delta\theta_{i}) \\ \sin(\Delta\theta_{i}) & \cos(\Delta\theta_{i}) \end{matrix}\right]\text{ }$ (24)

*4) Quantum rotation space transformation:* With$R(\theta_{i})$, the Q-bit of the individual is described:

(25)

## QDE

The pseudo-code of the basic QDE method [27] is presented in Algorithm 1. Let the QDE method contain a population set of Q-bit individuals, $Q(g)=(q_{1}^{g},q_{2}^{g},...,q_{NP}^{g})$ at the *g-*th generation, where *NP* and $q_{i}^{g}=\left[\left.\begin{matrix} \alpha_{i1}^{g} \\ \beta_{i1}^{g} \end{matrix}\right|\left.\begin{matrix} \alpha_{i2}^{g} \\ \beta_{i2}^{g} \end{matrix}\right|\left.\begin{matrix} \cdots\\ \cdots\end{matrix}\right|\begin{matrix} \alpha_{im}^{g} \\ \beta_{im}^{g} \end{matrix}\right]$ represent the population size and each Q-bit individual, respectively. The population set *Q* (*g*) needs to meet the quantum characteristics rule, shown in Appendix 5.1. The evolution procedure of the QDE method involves a quantum mutation stage, a quantum crossover stage, and a quantum selection stage.

| Algorithm 2: QDE algorithm (with DE/rand/1) | |
| --- | --- |
| 01: | Generate a uniformly distributed random initial population containing *NP* solutions, which include *NI* variables according to *α_i_^0^* = *α^min^* + *rand*[0, 1] * *(α^max^ - α^min^)* (*i*∈[1, *NI*]). Due to its DE/rand/1 scheme (shown in Eq. [25]), *NI* is equal to 3. We initialized the current iteration number *g* = 1 and defined *g* < *g_max_* (maximum iteration number), and set *F* and *CR to* ∈(0, 1]. |
| 02: | **while** *g* < *g_max_* |
| 03: | **for** *i*=1 to *NP* |
| 04: | Get three random indices *r_1_*, *r_2_*, and *r_3_* (*r_1_* ≠ *r_2_* ≠ *r_3_* ≠ *i*) // ***quantum mutation*** |
| 05: | *vec_i_^g^ = α_i1_^g^ + F* * *(α_i2_^g^ -α_i3_^g^)* // ***end quantum mutation*** |
| 06: | **if** *rand*[0, 1] ≤ *CR* // ***quantum crossover*** |
| 07: | *u_i_^g+1^* = *vec_i_^g^* |
| 08: | **else** |
| 09: | *u_i_^g+1^* = *α_i_^g^* |
| 10: | **end if** // ***end* *quantum crossover*** |
| 11: | **if** *f(u_i_^g^)* ≤ *f(α_i_^g^)* // ***quantum selection*** |
| 12: | *α_i_^g+1^ = u_i_^g^* |
| 13: | **else** |
| 14: | *α_i_^g+1^* =*α_i_^g^* |
| 15: | **end if** |
| 16: | **if** *f(u_i_^g+1^)* ≤ *f(α_i_^g+1^)* |
| 17: | *q_i_^g+1^* = *u_i_^g+1^* |
| 18: | **else** |
| 19: | *q_i_^g+1^* =*α_i_^g+1^* |
| 20: | **end if** // ***end* *quantum selection*** |
| 21: | **end for** |
| 22: | **end while** |

As shown in Algorithm 2, using the quantum initialization operation to obtain a quantum-based initial population set, QDE runs an evolution iteration such as quantum mutation, quantum crossover, and quantum selection until the termination requirement is satisfied. A detailed illustration of the key steps is shown below.

*1) Quantum mutation operator:* In the quantum mutation stage, the QDE model utilizes the mutation operator to obtain the mutant vector *vec_i_^g^*, which is expressed by each individual *α^g^_i_* at each generation *g*. Some well-known quantum mutation schemes are illustrated by,

$DE/rand/1:\text{ }vec_{i}^{g}=\alpha_{i1}^{g}+F\times(\alpha_{i2}^{g}-\alpha_{i3}^{g})$ (26)

$DE/rand/2:\text{ }vec_{i}^{g}=\alpha_{i1}^{g}+F\times(\alpha_{i2}^{g}-\alpha_{i3}^{g})+F\times(\alpha_{i4}^{g}-\alpha_{i5}^{g})$ (27)

$DE/best/1:\text{ }vec_{i}^{g}=\alpha_{best}^{g}+F\times(\alpha_{i1}^{g}-\alpha_{i2}^{g})$ (28)

and

$DE/best/2:\text{ }vec_{i}^{g}=\alpha_{i}^{g}+F\times(\alpha_{i1}^{g}-\alpha_{i}^{g})+F\times(\alpha_{i2}^{g}-\alpha_{i3}^{g})$ (29)

where *α^g^_best_* is the best individual at generation *g*. *i*, *i_1_*, *i_2_*, *i_3_*, *i_4_*, and *i_5_* are randomly selected within [1, *NI*], while none of them are equal to each other*.* The scaling factor *F* adjusts the mutation scale.

*2) Quantum crossover operator:* After mutation, the crossover operator is generated based on the quantum crossover operator scheme, as follows,

$u_{i}^{g+1}=\left\{\begin{matrix} vec_{i}^{g}\text{ }if(rand[0,1]\leq CR) \\ \alpha_{i}^{g}\text{ }otherwise \end{matrix}\right.$ (30)

where the crossover probability *CR* is within (0, 1]. Different values of *CR* have different influences on the QDE method. If the *CR* value is too large, the QDE method has fast convergence. By contrast, when the *CR* value becomes too small, the QDE method spends too much time finding the solution of the optimal global search.

*3) Quantum selection operator:* The greedy selection scheme adopted the QDE method as:

$\alpha_{i}^{g+1}=\left\{\begin{matrix} u_{i}^{g}\text{ }if\text{ }f(u_{i}^{g})<f(\alpha_{i}^{g}) \\ \alpha_{i}^{g}\text{ }otherwise \end{matrix}\right.$ (31)

$q_{i}^{g+1}=\left\{\begin{matrix} u_{i}^{\text{g+}1}\text{ }if\text{ }f(u_{i}^{g+1})<f(\alpha_{i}^{g+1}) \\ \alpha_{i}^{g+1}\text{ }otherwise \end{matrix}\right.$ (32)

## BPNN

Based on the former evaluation (Section 3.1.3), we adopted a three-layer structure comprising the input, hidden, and output layers, consisting of the number of neurons {*I_1_,…,I_I_*}, {*H_1_,..., H_H_*}, and {*O_1_,…, O_K_*}, respectively. This BPNN architecture involved forward- and backward-propagation steps. The forward-propagation step was adopted to compute the experimental outcome of each layer *Output* and the total error *E*. In addition, the backward-propagation step was adopted to optimize the model parameters, such as the model weight. We used *g* to represent the present iteration amount, and the learning rates of the model from the input-to-hidden layer and the hidden-to-output layer are represented as *LR_1_* and *LR_2_*, respectively, with both values within [0, 1].

Using the square error *E_i_* and the expected outputs of the output layer *eo*, the *E_Total_,* the forward-propagation step is computed by:

$E_{Total}=\sum_{i=1}^{n} E_{i}=\frac{1}{2}\sum_{i=1}^{n} (Output_{i}-eo_{i})^{2}$ (33)

In the back-propagation step, the weights between the layers were optimized according to the gradient descent (GD) technique [53] by:

$w_{2HK}(g+1)=w_{2HK}(g)-LR_{1}\times\frac{\partial E(w)}{\partial w_{2HK}(g)}$ (34)

and

$w_{1IH}(g+1)=w_{1IH}(g)-LR_{2}\times\frac{\partial E(w)}{\partial w_{1IH}(g)}$ (35)

## FBNNL

With its ability to minimize the error of the BPNN model, the gradient descent (GD) technique has been well used [54]. Furthermore, combing with the Caputo-type fractional gradient descent (CFGD) technique [33] and *L_2_* regularization, we used the FBNNL model [15] for training.

### Fractional-order backpropagation learning (FBL)

Due to its ability to optimize the model parameters in the backpropagation stage, we used the CFGD technique to replace the GD technique [55] in the FBL model. We defined the projection index *t*, fractional parameter *α*, gamma function $\Gamma$, sum function *F*(•) [15], and calibration parameter $cp=\min\{w_{1IH}(g),w_{2HK}(g)\}$, where *α* is within [0, 1]. According to Ref. [56], the Caputo derivative operator *Cap* (•) can be represented by:

$Cap(w_{1IH}(g))=\frac{1}{(1-\alpha)\times\Gamma(1-\alpha)}\times w_{2HK}(g)\times F^{'}(w_{1IH}(g)\times t_{H})\times t_{H}\times(w_{1IH}(g)-cp)^{1-\alpha}$ (36)

and

$Cap(w_{2HK}(g))=\frac{1}{(1-\alpha)\times\Gamma(1-\alpha)}\times g(w_{1IH}(g)\times t_{H})\times(w_{2HK}(g)-cp)^{1-\alpha}$ (37)

Based on the concept of the CFGD technique, both weight vectors *w_2HK_* and *w_1IH_* were modified to:

$w_{2HK}(g+1)=w_{2HK}(g)-LR_{1}\times C\text{a}p(w_{2IH}(g))\times\frac{\partial E(w)}{\partial w_{2HK}(g)}$ (38)

and

$w_{1IH}(g+1)=w_{1IH}(g)-LR_{2}\times Cap(w_{1IH}(g))\times\frac{\partial E(w)}{\partial w_{1IH}(g)}$ (39)

### *L_2_* regularization

Inheriting the satisfactory performance of resisting overfitting, *L_2_* regularization was added to the FBL model to build the FBNNL model. We defined the sum of the squares of the total weight vectors $\left\|w\right\|^{2}$ and regularization parameter $\lambda\geq0$, and the model error function *E_L2_* was optimized by:

$E_{L_{2}}=E_{Total}+\frac{\lambda}{2}\times\left\|w\right\|^{2}$ (40)

Based on Eq. (40), Eq. (38) and Eq. (39) were modified to:

$w_{2HK}(g+1)=w_{2HK}(g)-LR_{1}\times C\text{a}p(w_{2IH}(g))\times\frac{\partial E_{L_{2}}(w)}{\partial w_{2HK}(g)}$ (41)

and

$w_{1IH}(g+1)=w_{1IH}(g)-LR_{2}\times Cap(w_{1IH}(g))\times\frac{\partial E_{L_{2}}(w)}{\partial w_{1IH}(g)}$ (42)

## Clarification of equations of smooth the exponential contour

### Clarification of equation (20)

In this study, the FBNNL is adopted in the training procedure, where the output of the output layer is achieved at the forward-propagation process, and the model parameters are optimized at the back-propagation process. At the forward-propagation process, the Sigmoid activation function *h_1_* = 1 / (1 + *e^-x^*) and ELU activation function *h_2_* = (*e^x^* - 1) / 2 are adopted at the input-to-hidden and hidden-to-output layers, respectively. Here, we mainly indicate the process of achieving the equation (20).

We can obtain the input of the hidden layer *HL_I_*, shown as,

$HL_{I}=\sum_{i=1}^{H} tw_{1i}-a_{i};\text{ }i=1,2,..,H$ (43)

Then, the *h_1_* is adopted to achieve the output of the hidden layer *HL_O_*, represented as,

$HL_{O}=\frac{1}{1+e^{-HL_{I}}}=\frac{1}{1+e^{\sum_{i=1}^{H} -(tw_{1i}-a_{i})}};\text{ }i=1,2,..,H$ (44)

With *K* = 2, we can compute the input of the output layer *Output_I_*, indicated as,

(45)

The *h_2_* is adopted to achieve the output of the output layer *Output_O_*, calculated as,

(46)

Finally, the output neurons *Output*(•) are represented as,

(47)

### Clarification of equation (19)

In Ref. [57], a mathematical formula of the smooth organ curve is presented in which Sigmoid activation function is adopted at both input-to-hidden and hidden-to-output layers, respectively, represented as,

$fun(t)=\left(x(t),y(t)\right)=(\frac{1}{1+e^{Output_{o}(x)}},\frac{1}{1+e^{Output_{o}(y)}})=(\frac{1}{\sum_{j=1}^{K} \frac{1}{1+e^{\sum_{i=1}^{H} -(tw1_{i}a_{i})}}w_{2j,1}-b_{j,1}},\frac{1}{\sum_{j=1}^{K} \frac{1}{1+e^{\sum_{i=1}^{H} -(tw1_{i}-a_{i})}}w_{2j,2}-b_{j,2}})$ (48)

To deal with the gradient vanishing issue, the ELU activation function is adopted at the hidden-to-output layer [58], and *x*(*t*) and *y*(*t*) are represented as,

(49)

(50)

Based on Equations (48)~(50), our mathematical model (Equation (19)) of organ contours is indicated as,

$$f(t)=\left(x(t),y(t)\right)=fun(e^{Output_{o}(x)},e^{Output_{o}(y)})=\left(\frac{2\times Output(x(t))+1}{2\times Output(x(t))+2},\frac{2\times Output(y(t))+1}{2\times Output(y(t))+2}\right)$$
